# Supplementary material for: Getting More Out of Biomedical Documents with GATE's Full Lifecycle Open Source Text Analytics
Source: PLoS Comput Biol. 2013 Feb 7;9(2):e1002854. doi: 10.1371/journal.pcbi.1002854 (PMC3567135; doi:10.1371/journal.pcbi.1002854)
Supplement: Dataset S1 — GATE software. Dataset S1 bundled with this paper contains a distribution of GATE (or see http://gate.ac.uk/download/). (TGZ) [file pcbi.1002854.s001.tgz › plos-gate/plugins/Gazetteer_LKB/doc/usage.html]

The Large KB gazetteer - Usage


---

Last Published: 2009-10-17

---

##### Large KB Gazetteer

- Overview
- **Usage**
- Q & A
- GATE Compatibility
- GATE Gazetteer Feature Comparison
- Semantic Enrichment PR
- Samples

##### Developer Documentation

- Dictionary Lifecycle

- Required reading
- Basics
- Dictionary setup
- Additional dictionary configuration
- Processing Resource Configuration
- Run-time configuration

## Required reading

See at least the quick walkthrough in using GATE CREOLE plugins. The gazetteer is such plugin. You are probably familiar with RDF already if you have decided to use the Large KB gazetteer.

## Basics

- The Large KB Gazetteer is included with GATE starting with version 5.1. Download GATE at http://gate.ac.uk/download. The gazetteer requires GATE 5.1. See this if you need to use 5.0.
- There are other gazetteers in GATE that can process RDF. Select the right one for the job, using our comparison guide.
- To use the Large KB gazetteer, setup your dictionary first. The dictionary is a folder with some configuration files. Use the samples at *GATE\_HOME/plugins/lkb\_gazetteer/samples* as a guide or download a prebuilt dictionary from http://ontotext.com/kim/lkb\_gazetteer/dictionaries.
- Load *GATE\_HOME/plugins/lkb\_gazetteer* as a CREOLE plugin. See the GATE documentation for details.
- Create a new "Large KB Gazetteer" processing resource (PR). Put the folder of the dictionary you created in the dictionaryPath parameter. You can leave the rest of the parameters as defaults.
- Add the PR to your GATE application. The gazetteer doesn't require a tokenizer or the otput of any other processing resources.
- The gazetteer will create annotations with type Lookup and two features: "inst", which contains the URI of the ontology instance, and "class" which contains the URI of the ontology class that instance belongs to.

  See the detailed usage guide for details on setting up dictionaries and configuration.

## Dictionary setup

The dictionary is a folder with some configuration files. You can find samples at *GATE\_HOME/plugins/lkb\_gazetteer/samples*. Additionaly, prebuilt dictionaries can be downloaded from here.

Setting up your own dictionary is easy. You need to define your RDF ontology and then specify a SPARQL or SERQL query that will retrieve a subset of that ontology as a dictionary.

*config.ttl* is a Turtle RDF file which configures a local RDF ontology or connection to a remote Sesame RDF database.

If you want to see examples of how to use local RDF files, please check *samples/dictionary\_from\_local\_ontology/config.ttl*. The *Sesame repository configuration* section configures a local Ontotext SwiftOWLIM database that loads a list of RDF files. Simply create a list of your RDF files and re-use the rest of the configuration. The sample configuration support datasets with 10 000 000 triples with acceptable performance. For working with larger datasets, advanced users can substitute SwiftOWLIM with another Sesame RDF engine. In that case, make sure you add the necessary JARs to the list in *GATE\_HOME/plugins/lkb\_gazetteer/creole.xml*. For example, Ontotext BigOWL is an Sesame RDF engine that can load bilions of triples on desktop hardware.

Since any Sesame repository can be confgired in *config.ttl*, the Large KB gazetteer can extract dictionaries from **all** significant RDF databases. See the page on database compatibility for more information.

*query.txt* contains a SPARQL query. You can write any query you like, as long as its projection contains at least two columns in the following order: label and instance. As an option, you can also add a third column for the ontology class of the RDF entity. Below you can see a sample query, which creates a dictionary from the names and the unique identifiers of 10,000 entertainers in DbPedia.

```
PREFIX opencyc: <http://sw.opencyc.org/2008/06/10/concept/en/>
PREFIX rdfs: <http://www.w3.org/2000/01/rdf-schema#>

SELECT ?Name ?Person WHERE {
        ?Person a opencyc:Entertainer ; rdfs:label ?Name .           
    FILTER (lang(?Name) = "en") 
} LIMIT 10000
```

Try this query at the Linked Data Semantic Repository.

When you load the dictionary configuration in GATE for the first time, it creates a binary snapshot of the dictionary. Thereafter it will load only this binary snapshot. If the dictionary configuration is changed, the snapshot will be reinitialized automatically. For more information, please see the dictionary lifecycle specification

## Additional dictionary configuration

The config.ttl may contain additional dictionary configuration. Such configuration concerns only the initial loading of the dictionary from the RDF database. The options are still being determined and more will appear in future versions. They must be placed below the repository configuration section as attributes of a dictionary configuration. Here is a sample *config.ttl* file with additional configuration.

```
# Sesame configuration template for a (proxy for a) remote repository
#
@prefix rdfs: <http://www.w3.org/2000/01/rdf-schema#>.
@prefix rep: <http://www.openrdf.org/config/repository#>.
@prefix hr: <http://www.openrdf.org/config/repository/http#>.
@prefix lkbg: <http://www.ontotext.com/lkb_gazetteer#>.

[] a rep:Repository ;
   rep:repositoryImpl [
      rep:repositoryType "openrdf:HTTPRepository" ;
      hr:repositoryURL <http://ldsr.ontotext.com/openrdf-sesame/repositories/owlim>
   ];
   rep:repositoryID "owlim" ;
   rdfs:label "LDSR" .
   
[] a lkbg:DictionaryConfiguration ; 
  lkbg:caseSensitivity "CASE_INSENSITIVE" .
```

## Processing Resource Configuration

The following options can be set when the gazetteer PR is initialized:

- dictionaryPath - The dictionary folder described above.
- forceCaseSensitive - Whether the gazeteer should return case-sensitive matches regardless of the loaded dictionary.

## Run-time configuration

- annotationSetName - The annotation set, which will receive the generated lookup annotations.
- annotationLimit - The maximum number of the generated annotations. NULL or 0 for no limit. Setting limit of the number of the created annotations will reduce the memory consumption of GATE on large documents. Note that GATE documents consume gigabytes of memory if there are tens of thousands of annotations in the document. All PRs that create large number of annotations like the gazetteers and tokenizers may cause an Out Of Memory error on large texts. Setting that options limits the amount of memory that the gazetteer will use.

---

©
2009
Ontotext AD

---
